# Pomological and Biochemical Properties of Rosehip (*Rosa canina* L.) Genotypes

**DOI:** 10.1002/fsn3.72280

**Published:** 2026-10-08

**Authors:** Edanur Ertugrul, Erdal Aglar, Burhan Ozturk, Onur Tekin, Davut Alan, Umut Ates

**Affiliations:** ^1^ Department of Horticulture, Faculty of Agriculture Van Yüzüncü Yıl University Van Türkiye; ^2^ Design and Architecture, Department of Landscape Architecture Munzur University Faculty of Arts Tunceli Türkiye; ^3^ Department of Horticulture, Faculty of Agriculture Ordu University Ordu Türkiye; ^4^ Department of Horticulture, Faculty of Agriculture Sakarya University of Applied Sciences Sakarya Türkiye

**Keywords:** antioxidant activity, fruit color, fruit size, phenolic compounds

## Abstract

Rosehip ( *Rosa canina* L.) is an important wild fruit species valued for its high nutritional and functional properties, particularly its richness in vitamin C, phenolic compounds, and antioxidant activity. Despite the widespread natural distribution of rosehip in Türkiye, many local genetic resources remain insufficiently characterized, limiting their effective utilization in breeding, conservation, and cultivation programs. The present study was conducted to evaluate the morphological, pomological, and biochemical characteristics of naturally growing rosehip genotypes in the Central District of Tunceli and to identify superior genotypes with potential for future breeding and cultivation efforts. Ten rosehip genotypes collected from natural populations at altitudes ranging from 1178 to 1256 m were evaluated for fruit and seed characteristics, fruit color, soluble solids content, titratable acidity, vitamin C, phenolic compounds, flavonoids, anthocyanins, antioxidant activity, and organic acid composition. Significant differences were observed among genotypes for all examined traits. Fruit weight ranged from 1.00 to 1.81 g, soluble solids content from 45.40% to 51.90%, titratable acidity from 1.03% to 2.77%, and vitamin C content from 235.67 to 1126.30 mg/100 g. Total phenolic content, total flavonoid content, total monomeric anthocyanin content, FRAP, and DPPH antioxidant activity values also varied significantly among genotypes. Organic acid analysis revealed considerable variation, particularly in citric and malic acid contents. Principal component analysis explained 92.4% of the total variation and grouped the genotypes into two major clusters. Based on the overall evaluation, genotypes TK2, TK5, TK9, and TK10 exhibited superior performance for several desirable traits. The results demonstrate the substantial genetic diversity present in the natural rosehip populations of Tunceli and highlight their potential value for germplasm conservation, selection breeding, and sustainable cultivation programs.

## Graphical abstract

Overview of the characterization of 10 naturally growing *Rosa canina* L. genotypes (TK1–TK10) collected in Tunceli, Türkiye. The study evaluated fruit and seed morphology, fruit color, soluble solids, titratable acidity, vitamin C, phenolic and flavonoid compounds, anthocyanins, antioxidant activity (DPPH and FRAP), and organic acids, followed by multivariate analysis of 23 traits. Fruit weight ranged from 1.00 to 1.81 g and vitamin C from 235.67 to 1126.30 mg/100 g. PCA showed that 92.4% of the total variance was explained by six components. TK2, TK5, TK9, and TK10 were highlighted by the study as genotypes with superior performance across multiple characteristics.

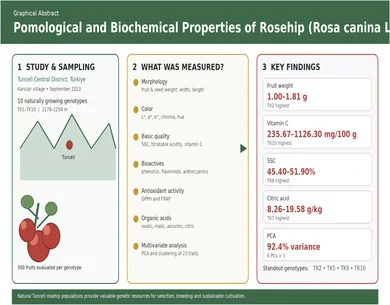

## 1 Introduction

Türkiye, one of the native lands of many cultivated fruit species in the world, is also among the homelands of rosehip (*Rosa* spp.), which grows wild and is widely utilized both for its fruit and plant (Ozbek 1977). Rosehip, belonging to the genus *Rosa* in the *Rosaceae* family's subfamily *Rosaoideae*, includes more than 100 taxa (Ercisli and Guleryuz 2005). Due to its resilience to extreme climatic and soil conditions, rosehip grows naturally across a wide range from Europe to Africa, and from Central and Western Asia to Russia (Nilson 1972; Ilisulu 1992).

In Türkiye, rosehip is found almost everywhere, but it is particularly abundant in the Central and Western Black Sea regions, such as Gumushane, Kastamonu, Corum, Amasya, and Tokat, as well as in Eastern Anatolia in provinces like Erzincan, Erzurum, Bitlis, Van, and Hakkâri, where harsh continental climates prevail (Dogan and Kazankaya 2006). Depending on the altitude, species, and variety, rosehip contains a high amount of vitamin C, ranging from 100 to 5300 mg per 100 g (Halasova and Jicinska 1988). Recently, it has gained popularity due to its health benefits, as it contains natural antioxidants, minerals, carotenoids, bioflavonoids, tocopherol, fruit acids, pectin, tannins, and amino acids (Cinar and Colakoglu 2005). Consequently, it has found wide application in industries such as food, pharmaceuticals, and cosmetics worldwide (Akyuz et al. 1996).

Despite the high value of rosehip, it has not received the necessary attention in Türkiye. Rosehip production in the country mostly relies on collecting wild species from nature. Although there are 27 species of rosehip in Turkiye (Ercisli and Guleryuz 2005), breeding efforts are limited to a small number of selection breeding studies. Unfortunately, the valuable genotypes identified through these selection efforts, as well as other genotypes that have not been studied, are at risk of being lost. This is because rosehip plants, with their thorny structure and shrub‐like form, occupy large areas and are often seen as undesirable in agricultural fields, leading to their eradication. As a result, many valuable genotypes are being lost. Therefore, it is crucial to identify these genotypes through selection studies and take measures to preserve them.

The province of Tunceli, with its mountainous terrain and rivers, has important microclimate zones that harbor a significant natural rosehip potential. However, no study has been conducted on this potential so far. The research we have planned will be the first study in the region, aiming to evaluate this valuable potential. With the completion of the study, the rosehip potential of the region will be examined. The area's topography and the presence of rivers create microclimates with diverse climatic characteristics, one of which is in the central district of Tunceli. This diversity enhances the importance of the region as a valuable source of plant genetic resources. The study aims to evaluate the genotypes of rosehip growing naturally in Tunceli, focusing on their fruit characteristics. By applying selection criteria, the goal is to identify superior genotypes with desirable traits.

## 2 Materials and Methods

### 2.1 Geographical and Climatic Characteristics of the Study Area

The province of Tunceli, located in Euphrates region of Eastern Anatolia, lies between 38°46′30″ N and 38°49′40″ E. It is bordered by the Munzur mountains and Karasu river to the north and west, the Bingöl mountains and Peri river to the east, and the Keban Dam Lake to the south. Tunceli's rugged terrain gradually descends from elevations of 3500–4000 m in the Munzur and Mercan mountains (part of the Taurus range) to 914 m toward the south. The province's average elevation is 945 m, and with an area of 7774 km^2^, it constitutes about 1% of Türkiye's total land area. Encompassed entirely within the Euphrates basin, the province is characterized by highlands enclosed by natural borders, where mountains have been shaped into elevated plateaus by erosion and rivers. Valleys are steep and narrow, with no plains forming at their bases. The topography of Tunceli consists of 70% mountains, 25% plateaus, and 5% plains. The climate of Tunceli features hot, dry summers and cold, rainy winters, with precipitation mainly occurring in autumn and spring as rain, and in winter as snow. Annual precipitation ranges between 550 and 1080 mm, with the least rainfall in summer, when strong northwesterly winds bring dryness. In winter, milder southwesterly winds dominate. According to meteorological data, the highest summer temperature in the southern regions can reach 42.2°C, whereas the lowest winter temperature in the northern mountainous areas can drop to around −26.6°C. The average annual temperature in Tunceli's central district is 12.5°C. The province's diverse topography, ranging from 3500 m mountains in the north to elevations as low as 700 m in the south, results in significant variations in climate elements such as precipitation, temperature, wind, and sunlight (Anonymous 2023). According to long‐term meteorological records, the study area is characterized by a continental climate with hot and dry summers and cold winters. The average annual temperature of the central district of Tunceli is approximately 12.5°C, while annual precipitation ranges between 550 and 1080 mm depending on altitude and topographic conditions. The substantial variation in elevation and microclimatic conditions within the region contributes to the diversity of environmental factors affecting rosehip growth, fruit development, and biochemical composition. Therefore, climatic conditions are considered one of the major factors influencing the phenotypic and biochemical variability observed among the evaluated genotypes.

### 2.2 Plant Material

This study was conducted in September 2023 in Karsılar village, Tunceli Central District. Within the wild rosehip populations naturally distributed in the region, individuals found in high concentrations were observed in detail. Ten individuals considered to be distinct from each other were selected as genotypes based on morphological characteristics such as fruit shape, fruit size, color, plant form, thorniness, and leaf structure. These individuals were identified based on visible morphological variation to represent natural variation. The selected genotypes were given the code “TK” (Tunceli Karşılar). Sampling was conducted in three replicates from each genotype, with 100 fruits per replicate. Fruit sampling was conducted when the fruit's hairy structures had fallen off, their color had darkened, consistent with the variety's characteristics, and they were easily picked by hand. These indicators were used as maturity indices. A total of 300 fruits were evaluated for each genotype. Taxonomic classification was made based on literature information and field observations, and the individuals used in the study were identified as *Rosa canina* L. However, herbarium sampling was not performed for definitive species identification.

### 2.3 Fruit Size

The fruit width refers to the widest part of the fruit's equatorial section, whereas the fruit length is measured as the distance between the stem cavity and the tip. These measurements were taken on 20 fruit samples using a digital caliper with 0.01 mm precision. Thirty fruits were randomly selected and weighed using a precision scale accurate to 0.01 g. The average fruit weight was calculated by dividing the total weight by the number of fruits (Celik 2007).

### 2.4 Number, Weight, Width, and Length of Seeds

Twenty fruits from each genotype were dissected, and the seeds were counted to determine the number of seeds per fruit. The seeds were weighed using a precision scale (0.01 g accuracy), and their width and length were measured using a digital caliper with 0.01 mm precision (Celik 2007).

### 2.5 Soluble Solid Content, Titratable Acidity and Vitamin C

In each replication, one slice was taken from 30 fruits and the homogenate obtained after being squeezed by an electric juicer was passed through a cheesecloth and the juice was obtained. SSC was measured with a digital refractometer (PAL‐1, Atago, USA) and expressed as a percentage. For titratable acidity determination, 10 mL of fruit juice was collected for each genotype and 10 mL of distilled water was added. The resulting solution was titrated with 0.1 mol/L sodium hydroxide (NaOH) until the pH reached 8.1. The pH was monitored during the titration using a calibrated pH meter. This method was performed using the potentiometric method, which provides more accurate results, rather than using a colored indicator. Titratable acidity was calculated based on the volume of NaOH consumed during titration, expressed as citric acid equivalents, and reported as g citric acid 100 mL^−1^ of fruit juice (Celik 2007). Reflectoquant plus 10 brand device (Merck RQflex plus 10, Türkiye) was used for the determination of vitamin C. After the fruit juice obtained for the SSC measurement was diluted 10‐fold with oxalic acid (5 g fruit juice sample, 50 mL oxalic acid), the ascorbic acid test kit was immersed in the diluted solution for 2 s, kept outside for 8 s to oxidize, and then placed into the test adapter of the Reflectoquant device for 5 s. The value read on the device was recorded and expressed as mg 100 g^−1^ (Ozturk et al. 2019).

### 2.6 Total Phenolic Compounds

Approximately 30 fruits per replicate were washed with distilled water and dried at room temperature. Seeds were removed, and the fruits were sliced and homogenized using a food blender. The homogenates (about 75–100 g) were transferred to Falcon tubes and stored at −80°C until analysis. Total phenolic content was determined using the Folin–Ciocalteu method as described by Ozturk et al. (2019). For each sample, 600 μL of fruit extract was mixed with 4.0 mL of distilled water, followed by 100 μL of Folin–Ciocalteu reagent and 300 μL of 2% sodium carbonate solution. After incubation at room temperature for 2 h, absorbance was measured at 760 nm using a UV–Vis spectrophotometer (Shimadzu, Japan). Quantification was based on a standard calibration curve constructed using gallic acid (0–500 mg·L^−1^). The equation of the standard curve was: 𝑦 = 0.0014𝑥 + 0.0392 (𝑅2 = 0.997). Results were expressed as mg gallic acid equivalents (GAE) per 100 g fresh weight (FW).

### 2.7 Total Flavonoid Content

Total flavonoids were measured following a modified method of Zhishen et al. (1999). A 600 μL sample of fruit extract was combined with 3.7 mL methanol and diluted to 4.3 mL. Then, 100 μL of 10% aluminum nitrate [(NO_3_)_3_] and 100 μL of 0.1 M ammonium acetate were added to reach a final volume of 4.5 mL. The mixture was incubated in the dark for 40 min at room temperature, and absorbance was measured at 415 nm. A standard calibration curve was prepared using quercetin (0–100 mg·L^−1^). The standard curve equation was: 𝑦 = 0.0049𝑥 + 0.0251 (𝑅2 = 0.998). The results were expressed as mg quercetin equivalents (QE) per 100 g FW.

### 2.8 Antioxidant Activity

#### 2.8.1 DPPH Free Radical Scavenging Assay

The antioxidant capacity of the samples was evaluated using the DPPH radical scavenging method based on Blois (1958), with modifications by Ozturk et al. (2019). A 500 μL extract sample was mixed with 2.5 mL of ethanol, and then 0.5 mL of 0.1 mM DPPH solution was added to bring the total volume to 4.0 mL. After vortexing for 1 min, the mixture was incubated in the dark for 30 min at room temperature. Absorbance was measured at 517 nm. The scavenging activity was calculated as a percentage of inhibition using the formula: % Inhibition = (A control−A Sample/A control) × 100. Results were quantified using a standard curve prepared with Trolox (0–300 μM). The standard curve equation was: 𝑦 = 0.2156𝑥 + 0.037 (𝑅2 = 0.996). Values were expressed as mmol Trolox equivalents (TE) per 100 g FW.

#### 2.8.2 Ferric Reducing Antioxidant Power (FRAP) Assay

The FRAP assay was conducted according to Ozturk et al. (2019). A 100 μL extract was mixed with 1.15 mL phosphate buffer (0.2 M, pH 6.7) and 1.25 mL potassium ferricyanide (1%). The mixture was incubated at 50°C for 20 min, cooled, and followed by the addition of 1.25 mL trichloroacetic acid (10%) and 0.25 mL ferric chloride (0.1%). Absorbance was recorded at 700 nm. The antioxidant capacity was calculated using a Trolox standard curve (0–250 μM), with the following equation: 𝑦 = 0.3012𝑥 + 0.029 (𝑅2 = 0.994). Results were expressed as mmol TE per 100 g FW.

### 2.9 Total Monomeric Anthocyanins

Total anthocyanin content was determined by the pH differential method as described by Giusti et al. (1999). Samples were diluted with two buffer systems (pH 1.0 and 4.5), and absorbances were read at 533 and 700 nm. The corrected absorbance (A) was calculated as: 𝐴 = (𝐴_533_−𝐴_700_)_pH 1.0_ − (𝐴_533_−𝐴_700_)_pH 4.5_. Anthocyanin content was calculated using cyanidin‐3‐glucoside (MW = 449.2 g·mol^−1^, ε = 26,900 L·mol^−1^·cm^−1^), and results were expressed as μg cyanidin‐3‐glucoside equivalents per g FW.

### 2.10 Organic Acids

Organic acids including ascorbic, oxalic, malic, and citric acids were quantified. A 15 g fruit sample was homogenized in 0.009 N H₂SO₄ (1:1, v/v) and shaken for 1 h. The mixture was centrifuged at 15,000 rpm for 15 min. The supernatant was filtered through a 0.45 μm membrane filter and a SEP‐PAK C18 cartridge prior to HPLC analysis. Analysis was performed using an Agilent HPLC 1100 series G1322A instrument equipped with an Aminex HPX‐87H column (300 mm × 7.8 mm, Bio‐Rad, USA), as per Bevilacqua and Califano (1989). Results were expressed in g·kg^−1^, except for fumaric acid, which was reported as mg·kg^−1^.

### 2.11 Statistical Analysis

The data, whose normal distribution suitability was determined by the Kolmogorov–Simirnov test and whose homogeneity was checked by the Levene test, were evaluated by analysis of variance. The significance level between applications was determined by Tukey's multiple comparison test. The significance level in interpreting the statistical results performed with the SAS package program (SAS 9.1 version, USA) is α = 5%. PCA analysis and biplot analysis, which explains the relationship between genotypes and examined traits, were performed using the JMP Pro 17 statistical package program.

## 3 Results and Discussion

### 3.1 Fruit and Seed Characteristics

Within the scope of the study, the fruit weight, fruit width, and fruit length values measured to determine the fruit size in genotypes identified at an altitude range of 1178–1256 m varied between 1.00 and 1.81 g, 10.07 and 13.02 mm, and 15.87 and 21.85 mm, respectively. Considering these values, it was observed that fruit size varied depending on the genotype, with significant differences among them. In terms of fruit weight, the TK2 genotype had the largest fruits, while the smallest fruits were harvested from the TK4 genotype (Table 1). As shown in Table 2, variations in seed weight, seed width, and seed length were also observed among genotypes. These values ranged from 0.27 g (TK4) to 0.64 g (TK5), 2.24 mm (TK2) to 3.00 mm (TK8), and 4.72 mm (TK6) to 6.58 mm (TK1), respectively. Fruit weight is one of the leading fruit quality criteria associated with fruit size and directly affects the market value of the product. Therefore, the selection and identification of ecotypes with high fruit weight are among the most important aspects for researchers working on fruit breeding (Sagbas 2023). Similar studies have yielded different results. For example, in a study on some fruit characteristics of rosehip types (*Rosa* sp.) selected from the Gevas and Ahlat regions, fruit width ranged from 12.26 to 17.73 mm, fruit length from 18.85 to 29.89 mm, fruit weight from 1.81 to 3.99 g, seed weight from 0.440 to 1.268 g, fruit flesh weight from 1.09 to 2.74 g, fruit flesh ratio from 52.43% to 79.61%, and seed ratio from 22.06% to 47.57% Balta and Cam (1996). Similarly, in rosehip genotypes naturally growing in the Tokat region, fruit weight ranged between 2.86 and 4.97 g, and the fruit flesh ratio varied from 57.22% to 77.38% (Gunes and Sen 2001). Kazankaya et al. (2001) reported that fruit weight in rosehip genotypes naturally growing in the Adilcevaz region varied from 1.2 to 3.62 g, with fruit flesh ratios ranging from 42.61% to 78.88% and seed counts between 13 and 48. Additionally, in the Van Lake basin, fruit weight ranged from 1.79 to 4.95 g, and the fruit flesh ratio was reported as 66.42 (Celik 2007). In the Amasya region, fruit weight was found to range from 1.37 to 3.04 g, with a fruit flesh ratio of 45.82%–79.47% (Dolek 2008). In the Muradiye region (Van), Yildiz and Celik (2011) reported fruit weight between 1.82 and 4.09 g, fruit length from 20.68 to 27.34 mm, fruit width from 12.97 to 15.74 mm, and fruit flesh ratio from 55.22% to 85.01%. In the Hakkâri region, Ekincialp et al. (2007) found fruit weight ranging between 1.52 and 3.92 g, with a fruit flesh ratio of 59.33%–76.69%. Beyhan et al. (2017) reported that the average fruit weight and flesh ratio in rosehip fruits varied between 2.95 and 4.02 g and between 59.3% and 78.4% (S‐10), respectively. It is thought that ecological and genetic factors may influence the variations in fruit size observed in these studies.

**TABLE 1:** Fruit size in rosehip genotypes naturally grown in Tunceli.

Genotype	Altitude (m)	Coordinate	Fruit width (mm)	Fruit length (mm)	Fruit weight (g)
TK1	1178	39°10′19″ N 39°26′40″ E	11.69 ± 0.43b–d	20.17 ± 0.64bc	1.63 ± 0.13ab
TK2	1174	39°10′17″ N 39°26′39″ E	12.87 ± 0.35ab	20.86 ± 0.44a–c	1.81 ± 0.11a
TK3	1232	39°10′28″ N 39°26′22″ E	10.85 ± 0.44c–e	19.31 ± 0.72cd	1.29 ± 0.12cd
TK4	1245	39°10′30″ N 39°26′22″ E	10.64 ± 0.34de	16.24 ± 0.31e	1.00 ± 0.05d
TK5	1245	39°10′31″ N 39°28′23″ E	12.61 ± 0.24ab	21.10 ± 0.56ab	1.78 ± 0.12a
TK6	1250	39°10′32″ N 39°26′23″ E	10.07 ± 0.53e	15.87 ± 0.27e	1.08 ± 0.08d
TK7	1256	39°10′35″ N 39°26′25″ E	11.84 ± 0.34a–c	21.85 ± 0.77a	1.62 ± 0.10ab
TK8	1263	39°10′36″ N 39°26′27″ E	10.75 ± 0.34c–e	18.32 ± 0.51d	1.44 ± 0.09bc
TK9	1250	39°10′33″ N 39°26′26″ E	13.02 ± 0.31a	18.53 ± 0.40d	1.74 ± 0.11ab
TK10	1246	39°10′31″ N 39°26′28″ E	12.39 ± 0.41ab	17.95 ± 0.47d	1.55 ± 0.13a–c

*Note:* Means in columns with the same letter do not differ according to Tukey's test at *p* < 0.05.

**TABLE 2:** Seed properties in rosehip genotypes naturally grown in Tunceli.

Genotype	Seed weight (g)	Seed width (mm)	Seed length (mm)
TK1	0.60 ± 0.05ab	2.54 ± 0.10bc	6.58 ± 0.25a
TK2	0.37 ± 0.03c–f	2.24 ± 0.14c	4.82 ± 0.31bc
TK3	0.34 ± 0.06d–f	2.50 ± 0.13bc	5.07 ± 0.21bc
TK4	0.27 ± 0.02f	2.72 ± 0.16ab	4.82 ± 0.13bc
TK5	0.64 ± 0.07a	2.80 ± 0.07ab	5.44 ± 0.13bc
TK6	0.31 ± 0.03ef	2.52 ± 0.10bc	4.72 ± 0.22c
TK7	0.51 ± 0.02a–c	2.49 ± 0.07bc	5.36 ± 0.25bc
TK8	0.41 ± 0.07c–f	3.00 ± 0.13a	5.51 ± 0.26b
TK9	0.50 ± 0.04a–d	2.58 ± 0.15bc	5.13 ± 0.26bc
TK10	0.47 ± 0.07b–e	2.55 ± 0.15bc	4.76 ± 0.15c

*Note:* Means in columns with the same letter do not differ according to Tukey's test at *p* < 0.05.

### 3.2 Fruit Color

Fruit color values (L*, a*, b*, chroma, and hue°) of the rosehip genotypes are presented in Table 3. Significant differences in color metrics were observed among genotypes. The highest L* value (42.01) was recorded for the TK10 genotype, whereas the lowest (34.04) was observed in TK1. The +a* value represents red and −a* green. The lowest a* value (31.27) was found in TK5, and the highest (40.16) in TK9. The +b* value indicates yellowness and ‐b* blueness. In this study, b* values ranged from −18.77 to 29.88, with the lowest in TK1 and the highest in TK10. Chroma, which indicates color vividness or dullness, varied significantly, ranging from 37.45 (TK1) to 49.22 (TK9). A lower hue° value indicates a shift toward red; the lowest hue° value (30.06) was measured in TK1, and the highest (39.66) in TK7. Similar studies have described rosehip fruit color as light red, dark red, matte red, and pomegranate red (Balta and Cam 1996). Sagbas (2023) reported chroma values for rosehip samples ranging between 53.04 and 60.71.

**TABLE 3:** Fruit color in rosehip genotypes naturally grown in Tunceli.

Genotype	L	a	b	C	Hue
TK1	34.04 ± 0.25e	32.40 ± 0.24b	18.77 ± 0.36c	37.45 ± 0.37c	30.06 ± 0.35c
TK2	38.36 ± 0.78bc	33.10 ± 1.86b	25.56 ± 1.52ab	41.93 ± 2.33bc	37.54 ± 0.88a
TK3	39.13 ± 0.37bc	32.22 ± 0.71b	27.76 ± 1.67a	41.60 ± 0.61bc	38.07 ± 0.25a
TK4	35.50 ± 0.34de	33.97 ± 0.38b	20.28 ± 0.26c	39.59 ± 0.28c	30.81 ± 0.53c
TK5	39.80 ± 1.51ab	31.27 ± 0.68b	25.58 ± 2.50ab	40.58 ± 1.77bc	38.95 ± 2.61a
TK6	36.87 ± 0.95 cd	33.40 ± 0.50b	21.88 ± 1.60bc	40.04 ± 1.31bc	33.10 ± 1.59bc
TK7	40.51 ± 1.36ab	33.87 ± 0.85b	28.30 ± 2.06a	44.28 ± 1.48b	39.66 ± 2.18a
TK8	36.65 ± 1.02c–e	34.40 ± 0.95b	21.22 ± 1.61bc	40.45 ± 1.67bc	31.54 ± 1.20bc
TK9	40.93 ± 0.39ab	40.16 ± 1.42a	28.26 ± 0.16a	49.22 ± 1.20a	35.22 ± 0.98ab
TK10	42.01 ± 0.59a	38.08 ± 1.14a	29.88 ± 0.72a	48.55 ± 1.27a	37.92 ± 0.57a

*Note:* Means in columns with the same letter do not differ according to Tukey's test at *p* < 0.05.

### 3.3 Soluble Solid Content, Titratable Acidity and Vitamin C

In the study, significant differences were observed among the genotypes in terms of SSC (soluble solid content), titratable acidity, and vitamin C content. The SSC values ranged from 45.40% (TK10) to 51.90% (TK8), while titratable acidity and vitamin C content varied between 1.03% (TK8) and 2.77% (TK2) and 235.67 (TK6) to 1126.30 mg/100 g (TK10), respectively (Table 4). The biochemical contents, such as SSC, titratable acidity, and vitamin C in rosehip, can vary depending on genetic and ecological factors as well as cultural practices. Indeed, selection studies on rosehip in different regions have revealed variations in chemical content. For example, Yildiz and Celik (2011) reported that in wild rosehip genotypes growing naturally in the Muradiye (Van) region, total dry matter content ranged from 42.98% to 55.88%, titratable acidity from 1.38% to 3.50%, and vitamin C content from 406.10 to 993.06 mg/100 g. Gunes (2011) stated that in rosehip genotypes from Tokat, total dry matter content ranged between 27.66% and 30.68%, while vitamin C content varied from 616.24 mg/100 g to 694.57 mg/100 g. In the rosehip genotypes found in the Hakkâri region, SSC values ranged from 14.25% to 27.50%, titratable acidity from 0.16% to 0.40%, and vitamin C content from 414.83 to 916.46 mg/100 g (Ekincialp et al. 2007). In the Hamur (Agrı) region, SSC values ranged from 9% to 32%, titratable acidity from 0.05% to 0.22%, and vitamin C content from 540 to 1315 mg/100 g (Beyhan et al. 2017). Similarly, Karakus and Bostan (2017) found that in rosehip genotypes growing naturally in Akıncılar, Sivas, vitamin C content varied between 438.79 and 766.13 mg/100 g, while total dry matter content ranged from 34.61% to 45.52%. Gunes and Dolek (2010) reported that in rosehip genotypes from Tokat, vitamin C content varied from 616.24 to 694.57 mg/100 g, total dry matter content from 27.66% to 30.68%, and SSC from 19.52% to 21.65%, suggesting that both cultivar and ecological factors may influence biochemical properties. In various studies conducted in different regions of Turkey, vitamin C content in rosehip was reported to range between 106 and 2712 mg/100 g FW (fresh weight) (Gunes and Sen 2001; Demir and Ozcan 2001; Ercisli 2007). Novajan et al. (2008) reported that vitamin C content in rosehip fruit ranged from 211 to 417.5 mg/100 g FW, whereas Jablonska‐Rys et al. (2009) and Rosu et al. (2011) reported higher values of 1252 mg/100 g FW and 643 mg/100 g FW, respectively. Sagbas (2023) found that vitamin C concentration in rosehip fruits varied between 507 and 621 mg/100 g. The reduction in vitamin C content in plants can result from environmental factors such as oxygen levels, the amount of light reaching the plant, changes in endogenous plant growth regulators, and temperature (Roman et al. 2013). Ascorbic acid (vitamin C) is a compound susceptible to oxidative degradation and can be easily degraded by oxidase enzymes, particularly when fruit tissues come into contact with high oxygen. This can lead to decreased ascorbic acid levels, particularly in regions where air temperatures rise during fruit ripening, oxygen concentration increases, or postharvest ventilation is inadequate. Oxidative stress conditions of this type can occasionally be observed in mountainous and semiarid regions like the Tunceli region, as well as in valleys with high diurnal temperature variations and limited air circulation, leading to low vitamin C values in some genotypes. Therefore, ascorbic acid levels observed may be associated not only with genetic differences but also with local microclimatic conditions. This finding can be considered an important finding in understanding the tolerance of genotypes to environmental stress conditions.

**TABLE 4:** Soluble solids content, titratable acidity and vitamin C in rosehip genotypes naturally grown in Tunceli.

Genotype	Soluble solids content (%)	Titratable acidity (%)	Vitamin C mg/100 g
TK1	51.47 ± 0.12a	1.73 ± 0.03de	634.00e
TK2	48.53 ± 0.12c	2.77 ± 0.03a	624.67e
TK3	49.93 ± 0.43b	1.93 ± 0.03d	589.00f
TK4	50.13 ± 0.09b	2.53 ± 0.03b	607.33ef
TK5	48.13 ± 0.28cd	2.37 ± 0.09bc	921.00b
TK6	47.63 ± 0.59d	2.27 ± 0.03c	435.67g
TK7	45.50 ± 0.06e	1.87 ± 0.03d	762.30c
TK8	51.90 ± 0.06a	1.03 ± 0.09f	621.30e
TK9	51.43 ± 0.26a	1.53 ± 0.19e	713.70d
TK10	45.40 ± 0.29e	2.30 ± 0.06bc	1126.30a

*Note:* Means in columns with the same letter do not differ according to Tukey's test at *p* < 0.05.

### 3.4 Total Phenolics, Flavonoids, Anthocyanins and Antioxidant Activity

The content and concentration of bioactive compounds such as vitamins, minerals, phenolics, flavonoids, organic acids, and anthocyanins in fruits can vary depending on species, variety, and ecological conditions (Serra et al. 2011). Rosehip is a fruit species rich in numerous bioactive compounds essential for human nutrition and health, including sugars, organic acids, pectins, flavonoids, tannins, carotenoids, fatty acids, vitamins (especially vitamin C), and macro‐ and microelements (Demir and Ozcan 2001; Orhan et al. 2009). The content and concentration of these compounds also change according to genetic and ecological factors. Roman et al. (2013) reported that the total polyphenol content in rosehip fruits ranged from 326.5 to 575.0 mg GAE/100 g. Similarly, Beyhan et al. (2017) found variations in bioactive compounds such as total phenolics, total flavonoids, total carotenoids, and antioxidant activity depending on the genotype, with total phenolic content ranging from 340 to 464 mg GAE/100 g. Other studies have reported similar ranges: Nilsson (1997) and Yoo et al. (2008) recorded total phenolic content between 326 and 818 mg GAE/100 g in rosehip fruits. In the present study, total phenolic content, which varied depending on genotype, was determined to be between 38.50 mg GAE/100 g (TK7) and 95.29 mg GAE/100 g (TK2). The total flavonoid content and total monomeric anthocyanin values ranged from 18.00 g QE/kg (TK7) to 68.40 g QE/kg (TK10) and from 188.66 mg cyanidin‐3‐glucoside/kg (TK6) to 2998.90 mg cyanidin‐3‐glucoside/kg (TK7), respectively (Table 5). Significant differences among genotypes were also observed in terms of antioxidant activity, with FRAP and DPPH values ranging from 655.27 mmol TE/kg (TK1) to 2098.20 μmol TE/kg (TK7) and from 124.90 mmol TE/kg (TK8) to 154.70 mmol TE/kg (TK10), indicating relatively high antioxidant activity compared to previous studies (Table 6). It is known that phenolic compounds, such as anthocyanins, flavonoids, and phenolic acids, are responsible for antioxidant activity in fruits, and fruits with high phenolic content typically exhibit stronger antioxidant activity. Ersoy (2019) in a study evaluating fruit properties of different *Rosa pimpinellifolia* genotypes, reported that total anthocyanin content ranged from 3.45 to 4.31 mg/100 g, total phenolic content from 971 to 1138 mg GAE/100 g, and antioxidant activity from 9.23 to 12.17 mmol Fe(II)/g. Murathan et al. (2016) found that the total anthocyanin content of various rosehip species ranged from 2.43 to 3.72 mg/100 g, total phenolic content from 1081 to 6298 mg GAE/100 g, and antioxidant capacity (measured by FRAP) from 10.04 to 97.95 mmol Trolox/g, with vitamin C content ranging from 24.93 to 754.48 mg/100 g. Guerrero et al. (2010) analyzing the bioactive components of different blackberry fruits, reported that anthocyanin content in rosehip was 0.38 mg/100 g, while total phenolic content was 145.7 mg/100 g. In a characterization study of 25 rosehip genotypes, Demir et al. (2021) found antioxidant activity between 39.510 and 72.673 mmol/kg, total flavonoid content from 287.80 to 1686.20 mg QE/kg, and total phenolic content from 38.519 to 79.080 mg GAE/kg. Additionally, Paunovic et al. (2019) reported total phenolic content of 90.51 mg GAE, total flavonoid content of 38.52 mg QE, and antioxidant activity of 0.32 mM TE in rosehip fruits.

**TABLE 5:** Total phenolics, total flavonoid, and total monomeric anthocyanin in rosehip genotypes naturally grown in Tunceli.

Genotype	Total phenolics	Total flavonoid	Total monomeric anthocyanin
	g GAE kg^−1^	g QE kg^−1^	mg siyanidin 3‐glikozit kg^−1^
TK1	74.26a–c	41.65d	1221.46bc
TK2	95.29a	36.10e	1133.18bc
TK3	84.09ab	48.13c	814.27c
TK4	78.49ab	45.68cd	1330.63bc
TK5	69.35a–c	55.01b	1002.94bc
TK6	72.79a–c	31.34f	188.67d
TK7	38.50c	18.00h	705.00c
TK8	61.20a–c	25.00g	2998.90a
TK9	52.70bc	29.80f	2290.00ab
TK10	75.50ab	68.40a	1439.90bc

*Note:* Means in columns with the same letter do not differ according to Tukey's test at *p* < 0.05.

**TABLE 6:** Antioxidant activity in rosehip genotypes naturally grown in Tunceli.

Genotype	DPPH, mmol TE kg^−1^	FRAP, mmol TE kg^−1^
TK1	135.73ef	655.27c
TK2	153.39ab	1853.42a
TK3	142.71c–e	1238.50b
TK4	133.73f	1222.88b
TK5	143.71cd	959.28bc
TK6	144.51cd	810.49bc
TK7	138.00d–f	2098.20a
TK8	124.90g	754.90bc
TK9	146.20bc	880.30bc
TK10	154.70a	1114.00bc

*Note:* Means in columns with the same letter do not differ according to Tukey's test at *p* < 0.05.

### 3.5 Organic Acids

In the study conducted on rosehip genotypes growing naturally in Tunceli, significant variations were detected in terms of organic acid content. The highest oxalic acid level was measured as 0.56 g/kg in the TK1 genotype and this value was found to be statistically significantly higher compared to other genotypes; the lowest value was recorded as 0.13 g/kg in TK10. Although the amount of oxalic acid is generally low, its high level in the TK1 genotype is important due to its toxicological effects. The richest genotype in terms of malic acid content was TK2, which stood out among all genotypes with 3.75 g/kg. On the other hand, malic acid content was found to be quite low in the TK3 and TK7 genotypes, 0.95 and 1.01 g/kg, respectively. Malic acid is one of the acids with the widest variation among genotypes and is known to contribute significantly to the taste profile. Although ascorbic acid (vitamin C) content was generally low, the TK10 genotype stood out in this respect and had by far the highest ascorbic acid content with 0.22 g/kg. TK8 was the genotype with the lowest ascorbic acid content with only 0.02 g/kg. Ascorbic acid is of great importance in terms of nutritional value and TK10 stands out in this context. Citric acid was found in high concentrations in all genotypes and reached the highest values by far compared to all other organic acid types. Although it was measured at high levels such as 19.58 g/kg in TK7 and 19.27 g/kg in TK1, it was found to have the lowest value of 8.26 g/kg in TK8 genotype. It is known that citric acid plays an important role in the food industry as both a flavor enhancer and a natural preservative (Table 7). These findings show both similarities and some differences with the results previously reported in the literature by Ercisli (2007), Adamczak et al. (2012) and Barros et al. (2010). For example, Ercisli (2007) reported the ascorbic acid content in rosehip species as 727–943 mg/100 g, while lower values were obtained in this study; this may be due to differences in methods, fruit maturity level or environmental conditions. On the other hand, Barros et al. (2010) also reported the ascorbic acid level in *Rosa canina* fruits as 68.04 mg/100 g DM and similar levels were observed with this study. In addition, Adamczak et al. (2012) reported citric acid levels as 3.16 mg/100 g on average, but higher citric acid levels were recorded in Tunceli genotypes in this study. It was emphasized by Adamczak et al. (2012) that organic acids provide positive effects not only on fruit aroma and flavor, but also on antioxidant effect, protection and stability of vitamin C. The study results are consistent with the findings reported by Murathan et al. (2016), Yildiz and Alpaslan (2012) and Fascella et al. (2019). In conclusion, there are significant variations among the phytochemical components of rosehip genotypes, and these variations provide valuable information for the selection of both health‐important compounds and elements contributing to food quality.

**TABLE 7:** Organic acids in rosehip genotypes naturally grown in Tunceli.

Genotype	Oxalic acid, g kg^−1^	Malic acid, g kg^−1^	Ascorbic acid, g kg^−1^	Citric acid, g kg^−1^
TK1	0.56a	2.37d	0.04cde	19.27a
TK2	0.30b	3.75a	0.10b	12.64b
TK3	0.26bc	0.95g	0.05cd	9.91e
TK4	0.30b	3.17b	0.05cde	10.44cde
TK5	0.23bc	3.00bc	0.06c	10.64cd
TK6	0.20cd	2.78c	0.03de	10.92c
TK7	0.25bc	1.01g	0.04cde	19.58a
TK8	0.18cd	1.66f	0.02e	8.26f
TK9	0.25bc	2.01e	0.04cde	10.10de
TK10	0.13d	1.67f	0.22a	12.07b

*Note:* Means in columns with the same letter do not differ according to Tukey's test at *p* < 0.05.

### 3.6 Principal Component Analysis (PCA)

Principal Component Analysis (PCA) was conducted based on 23 morphological and biochemical traits. Among the 23 extracted components, 6 principal components (PCs) exhibited eigenvalues > 1.0 and collectively accounted for 92.4% of the total variance observed among genotypes. The first two components alone explained 52.1% of the cumulative variance, whereas the first 3, 4, and 5 components accounted for 69.0%, 80.1%, and 87.1% of the total variance, respectively. PC1, which accounted for the largest proportion of variation (33.1%), was primarily associated with fruit width, color parameters (L, b, C*, hue), soluble solids content, vitamin C, DPPH, and ascorbic acid. PC2, explaining 19.0% of the variation, was related to fruit length, fruit weight, seed weight, seed length, titratable acidity, and total phenolics. PC3, responsible for 16.9% of the variation, was linked with seed width, a* value, total monomeric anthocyanin, oxalic acid, and citric acid. PC4, explaining 11.0% of the total variance, was associated with total flavonoids, FRAP, and malic acid. PC5 and PC6, which accounted for 6.9% and 5.3% of the total variation, were related to total flavonoids and citric acid, respectively. When examining the major contributing variables for each component, the b color value (0.92) was found to be the dominant trait for PC1. Seed weight (0.78) was the most influential trait in PC2, while citric acid significantly contributed to both PC3 (0.67) and PC6 (−0.48). Total flavonoids had high loadings in both PC4 (0.68) and PC5 (−0.45). These results suggest that rosehip genotypes exhibit high correlations particularly across the traits represented by PC1 and PC2 (Table 8; Figure 1). Demir et al. (2021) examined certain morphological and biochemical characteristics of 25 rosehip genotypes cultivated in the Ordu and Kayseri regions and reported that three PCs explained 98.57% of the total variation, emphasizing the potential presence of both positive and negative correlations among the evaluated traits.

**TABLE 8:** Principal component analysis of rosehip genotypes.

Characters	PC1	PC2	PC3	PC4	PC5	PC6
Fruit width	**0.70311**	0.40534	0.19962	0.37385	0.36058	0.11837
Fruit length	0.37971	**0.60402**	0.58334	−0.11622	−0.00011	0.32250
Fruit weight	0.53070	**0.63548**	0.30040	0.26724	0.27298	0.22618
Seed weight	0.26949	**0.78284**	0.27986	0.32073	−0.23108	0.02798
Seed width	−0.43505	0.30041	**−0.57228**	0.19266	−0.43421	0.33683
Seed length	−0.42062	**0.75614**	0.37851	0.21335	−0.16729	−0.14780
L	**0.92171**	0.10252	−0.29884	−0.13529	−0.02968	0.10284
a	0.40476	0.14801	**−0.66244**	0.13017	0.39412	−0.42525
b	**0.92197**	0.06738	−0.19683	−0.19922	0.02132	0.04387
C	**0.78520**	0.14373	−0.49411	−0.02852	0.24530	−0.22675
Hue	**0.86433**	0.02934	0.14729	−0.27438	−0.13350	0.31827
Soluble solids content	**−0.70555**	0.27068	−0.19508	0.32203	0.39030	0.12018
Titratable acidity	0.35251	**−0.71382**	0.50478	0.14929	0.00241	0.08031
Vitamin C	**0.74427**	0.24808	−0.11944	0.39025	−0.39766	−0.03558
Total phenolics	−0.08647	**−0.67119**	0.32305	0.49605	0.10669	0.11878
Total flavonoids	0.35588	−0.34845	0.02913	**0.68811**	**−0.45655**	−0.08081
Total monomeric anthocyanin	−0.16540	0.47453	**−0.62719**	0.33696	0.28082	0.16254
DPPH	**0.79227**	−0.37179	0.19720	0.21882	0.22476	−0.13062
FRAP	0.50315	−0.10571	0.38131	**−0.57749**	0.16766	0.14837
Oxalic acid	−0.47870	0.29943	**0.66518**	0.22208	0.17812	−0.31564
Malic acid	−0.17527	−0.43370	0.37987	**0.45672**	0.35862	0.26963
Ascorbic acid	**0.73417**	−0.25362	−0.04518	0.41500	−0.19326	−0.26502
Citric acid	0.08326	0.42192	**0.67832**	−0.25766	−0.09797	**−0.48760**
Eigen value	7.6	4.3	3.8	2.5	1.6	1.2
% variance	33.1	19.0	16.9	11.0	6.9	5.3
Cumulative variance	33.0	52.1	69.0	80.1	87.1	92.4

*Note:* Factor loading ≥ 45.6 are bold.

**FIGURE 1:**
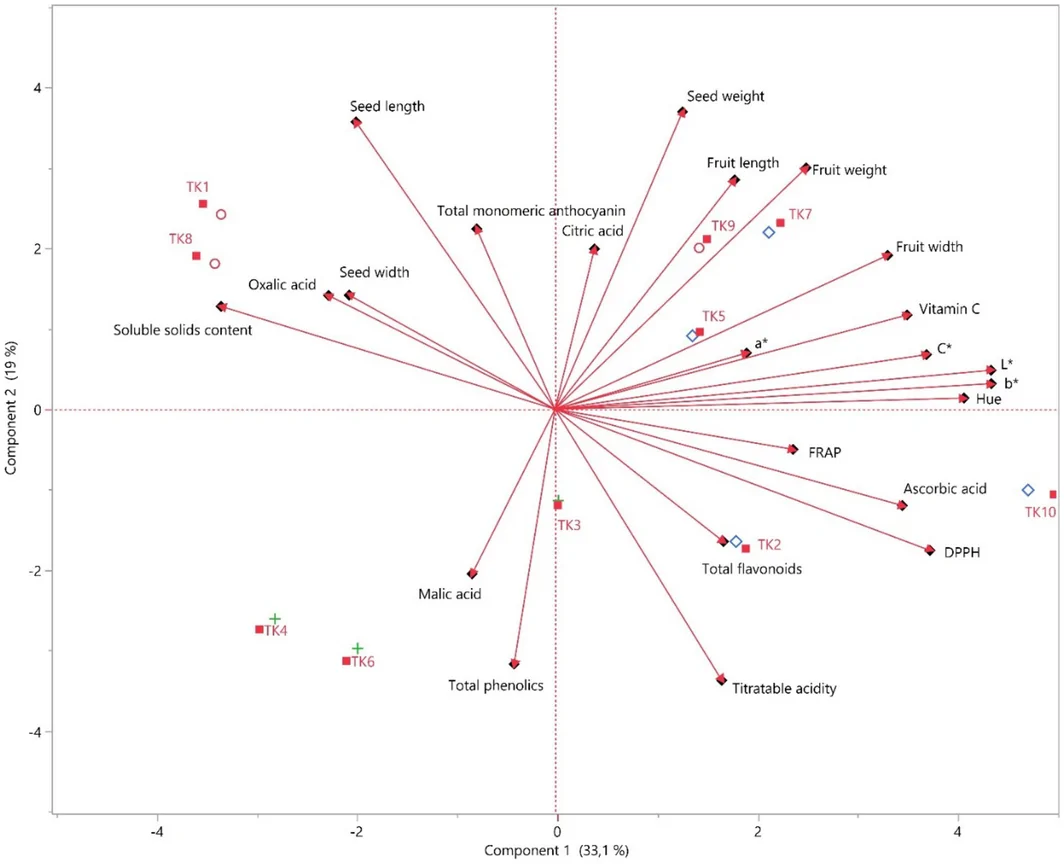
Component plot of the first two principal components (PCI and PC2) in the rosehip genotypes.

A dendrogram analysis was performed using a total of 23 traits, including fruit and seed characteristics, color characteristics, soluble solids content, titratable acidity, vitamin C, total phenolics, flavonoids, anthocyanins, antioxidant activity, oxalic acid, malic acid, ascorbic acid, and citric acid, to examine the rosehip genotypes. The analysis resulted in the formation of two main groups (A and B). The first main group (A) was further divided into two subgroups (A‐1 and A‐2), whereas no further distinction was observed within the second group (B). Specifically, the A group consisted of 6 genotypes in total: three genotypes (TK1, TK8, and TK9) in subgroup A‐1, and three genotypes (TK3, TK4, and TK6) in subgroup A‐2. The B group comprised four genotypes: TK2, TK5, TK7, and TK10. In the A group, TK1 in subgroup A‐1 was characterized by seed length and oxalic acid, while TK8 was notably associated with seed width, soluble solids content, and total monomeric anthocyanin. TK9 showed significant results in fruit width, a, and C values. Within subgroup A‐2, TK3 was distinguished by total phenolics, TK4 by malic acid, and TK6 did not exhibit any particular associations with the examined traits. In the B group, TK2 showed major values for fruit width, fruit weight, titratable acidity, total phenolics, and malic acid, while TK5 was characterized solely by its seed weight. TK7 stood out with major values for fruit length, hue, FRAP, and citric acid, and TK10 was notable for its L, b, vitamin C, total flavonoids, DPPH, and ascorbic acid levels (Figure 2).

**FIGURE 2:**
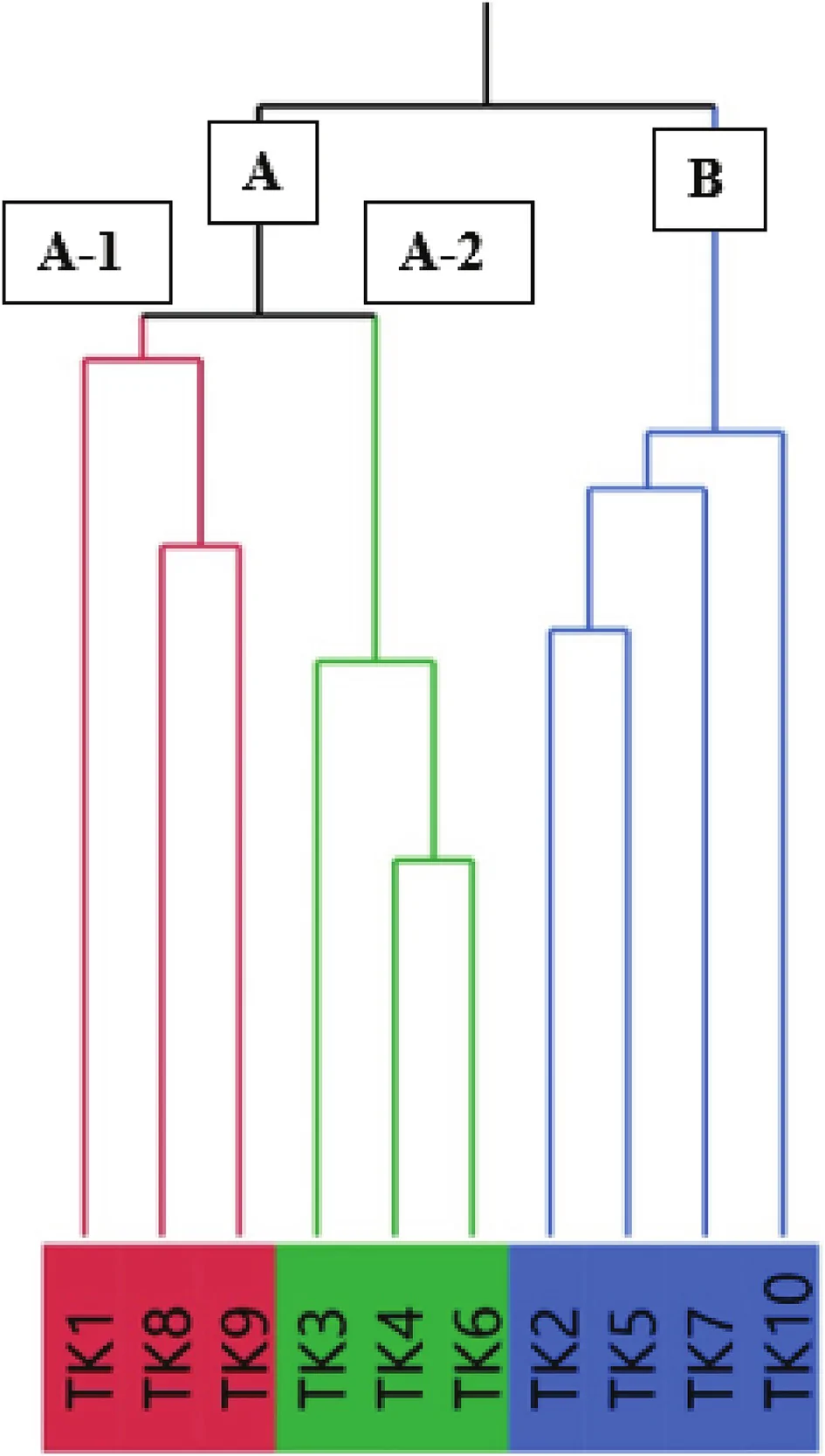
Dendrogram grouping of rosehip genotypes based on bioactive compounds.

## 4 Conclusion

This study revealed significant morphological and biochemical variability among naturally growing rosehip ( *Rosa canina* L.) genotypes in the central district of Tunceli. Fruit size, color characteristics, soluble solids content, titratable acidity, vitamin C, phenolic compounds, flavonoids, anthocyanins, antioxidant activity, and organic acid composition differed significantly among genotypes. Among the evaluated materials, TK2, TK5, TK9, and TK10 exhibited superior performance for several desirable traits.

The results demonstrate that the natural rosehip populations of Tunceli represent valuable genetic resources with considerable potential for germplasm conservation, selection breeding, and future cultivation programs. Further studies involving molecular characterization and multi‐location evaluations are recommended to support the effective utilization and conservation of these genetic resources.

## Author Contributions

**Umut Ates:** conceptualization, investigation, methodology, validation, visualization, writing – original draft. **Edanur Ertugrul:** methodology, investigation, validation, conceptualization, writing – review and editing. **Onur Tekin:** conceptualization, investigation, writing – original draft, methodology, validation, visualization. **Burhan Ozturk:** methodology, investigation, conceptualization, validation, writing – original draft, visualization. **Erdal Aglar:** conceptualization, methodology, writing – review and editing, validation, investigation. **Davut Alan:** conceptualization, investigation, writing – original draft, methodology, validation, visualization.

## Funding

The study was supported by the project numbered 1919B012301457 of The Turkey Scientific and Technological Research Council.

## Ethics Statement

The plant material used in this study consisted of rose hip (*Rosa* spp.) genotypes naturally growing in the central district of Tunceli, Türkiye. Plant samples were collected from publicly accessible areas in accordance with local and national regulations governing the use of natural resources. No protected, endangered, or legally restricted plant species were involved in the study, and no sampling was conducted within protected conservation areas requiring special permits. Therefore, no specific collection permit or ethical approval was required for the collection of plant materials. All research activities were carried out in compliance with applicable institutional, national, and environmental regulations. The collection and use of plant materials complied with relevant biodiversity and conservation guidelines, and the study did not involve genetically modified organisms, human participants, human tissues, animals, or animal‐derived materials. Accordingly, approval from an Institutional Review Board or Animal Ethics Committee was not required.

## Consent

The authors have nothing to report.

## Conflicts of Interest

The authors declare no conflicts of interest.

## Data Availability

All data generated or analyzed during this study are included in this published article.
